# International prevalence of adverse drug events in hospitals: an analysis of routine data from England, Germany, and the USA

**DOI:** 10.1186/1472-6963-14-125

**Published:** 2014-03-13

**Authors:** Jürgen Stausberg

**Affiliations:** 1 Institut für Medizinische Informationsverarbeitung, Biometrie und Epidemiologie (IBE), Ludwig-Maximilians-Universität München, Marchioninistraße 15, 81377 München, Germany

**Keywords:** Adverse drug event, Adverse drug reaction reporting system, Hospitals, International classification of diseases, Medication errors, Routine data

## Abstract

**Background:**

Adverse drug events (ADEs) are frequent in hospitals, occurring either in patients before admission or as a nosocomial event, and either as a drug reaction or as a consequence of a medication error. Routine data primarily recorded for reimbursement purposes are increasingly being used on a national level both in pharmacoepidemiological studies and in trigger tools. The aim of this study was to compare the prevalence rates of coded ADEs in hospitals on a transnational level.

**Methods:**

Hospital data for England and the USA were obtained for the fiscal or calendar year 2006. German data for 2006 were accessed via teleprocessing with the Federal Statistical Office. The datasets from England and the USA were adapted to the German data. About 6 million (England), 7 million (USA), and 16 million (Germany) inpatients could be included. ADEs were identified through a list of codes used in the national diagnosis classifications.

**Results:**

The overall prevalence rate (and 95% confidence interval, CI) of coded ADEs was 3.22% (3.20–3.23%) for England, 4.78% (4.73–4.83%) for Germany, and 5.64% (5.63–5.66%) for the USA. Most of the English ADE cases occurred in patients admitted as emergency. A non-surgical status and a longer length of stay were consistently associated with the occurrence of an ADE. Enterocolitis caused by Clostridium difficile was the most frequent ADE in all countries.

**Conclusions:**

According to routine data, the overall ADE prevalence rates for England, Germany, and the USA are different. However, the differences are narrower than those determined from the rates of ADEs or adverse drug reactions inferred from prospective or retrospective pharmacoepidemiological studies. Since the ADEs in the countries examined in this study share several characteristics, the use of routine data for transnational research on ADEs is feasible.

## Background

Adverse drug events (ADEs) can be defined as “harm caused by the use of a drug” [1], which implies that they are identifiable through their associated symptoms and diseases. Therefore, upon initial consideration, the coding of ADEs using the International Classification of Diseases and Health Related Problems (ICD) should be possible, because the ICD thoroughly covers diseases and symptoms. However, it is also possible that coding circumstances and standards hamper the use of ICD codes for ADEs [2]. Nonetheless, there is a growing body of literature on pharmacovigilance employing ICD-coded diseases and symptoms [3-5]. The codes could also be used in the timely detection of ADEs, especially when evaluated in combination with other information such as lab values in trigger tools [6].

However, the definition of an ADE as “harm caused by the use of a drug” [1] excludes a few relevant issues related to medication. On the one hand, medication errors that do not cause harm are excluded, as are near-miss events. Those situations do not interfere with the use of ICD codes because they do not lead to symptoms or diseases. On the other hand, an unintended low dose or a missing prescription of a drug could cause harm related to the inappropriate treatment of a patient’s condition. In such cases, ICD codes might indicate the harm as ongoing or as a deteriorating pathological state of the patient. The complex and manifold settings considered in patient safety and pharmacovigilance are reflected in the diversity of terms and definitions used to describe them [7].

ADEs are a burden on healthcare systems because of the resources needed to diagnose and treat the symptoms and diseases caused by them. From the perspective of society, these costs are unnecessary, regardless of the point of origin. Thus, ADEs present on admission and nosocomial ADEs should be grouped together to estimate the cost of ADEs in inpatients. A microcosting study in Germany used routine data to extrapolate the annual total costs, reporting a price tag of about 1 billion Euros [8]. This amount is in line with the one published for preventable ADEs in the USA, around 2 billion USD [9].

It seems reasonable to estimate the burden of ADEs arising from the inpatient sector by using routine data to calculate the frequencies of ICD-coded diagnoses. Routine data are generated because of regulations in reimbursement systems that apply diagnosis-related groups (DRGs) and they are available at low cost in many developed countries. A report of the Organisation for Economic Co-operation and Development (OECD) uses such data to compare the rates of the Agency for Healthcare Research and Quality (AHRQ) Patient Safety Indicators, provided by participating countries to enable a transnational comparison [10]. For example, 19 countries contributed data for the indicator “Foreign Body Left During Procedure”. All but three of those countries apply DRGs for reimbursement, and all but one apply the ICD system for the coding of diagnoses. Thus, it should also be possible to use routine data for a transnational comparison of ADEs. In the following, we present the results of a study on the frequency of ADEs in England, Germany, and the USA. These countries were chosen because of the availability of data and/or the existence of previous relevant publications. The aims of this study were to elaborate the feasibility of transnational comparisons using routine data and, by applying the same approach, to compare routine-data-based ADE rates between different countries.

## Methods

### Definitions

In this study, an ADE was defined as an injury resulting from a medical intervention related to a drug and thus included both adverse drug reactions (ADRs) and the consequences of medication errors [11]. ADEs related to the omission of a medication or to a medication dose that was too low were not explicitly covered by this definition. ADEs present at the time of admission and those occurring during a hospital stay were both considered. Accordingly, the results reflect hospitals’ burdens of ADEs independent of the question of responsibility. In the international literature in which ICD codes were applied in the identification of ADEs, the terms ADR and ADE are used inconsistently [7]. Since many ICD codes do not precisely distinguish between ADRs and the consequences of medication errors, the term ADE is preferably used throughout this paper.

### Datasets

The National Health Service Information Centre for Health and Social Care (NHS Information Centre) offers datasets for the different sectors of the English health care system. The Hospital Episode Statistics (HES) includes all inpatients served by “acute hospitals, primary care trusts and mental health trusts” [12]. It covers a financial year extending from April to March, with data available for every financial year from 1989/1990 onwards. HES data are collected monthly. The observational unit is the episode, defined as the period of time that a patient is under the care of one consultant. Episodes can be aggregated to describe either an inpatient stay or a particular patient by using different identifiers or service-related data elements. Diagnoses are coded using the ICD-10, and procedures by a national procedure classification. A maximum of 20 diagnosis codes and 24 procedure codes are allowed per episode. We obtained the raw data for the financial year 2006/2007 (1^st^ April 2006 to 31^st^ March 2007). These raw data were representative of 15,804,643 episodes. Inpatients admitted before or discharged after the financial year were included as long as there was at least one episode that overlapped with that financial year.

The AHRQ maintains the National Inpatient Sample (NIS) as part of the Healthcare Cost and Utilization Project [13]. The NIS has been available yearly since 1988. Information on inpatient stays consists of clinical and resource use aspects, available from discharge abstracts. The NIS draws information from the 20% of hospitals in the USA that are stratified for region, location, teaching status, bed size, and ownership. The inclusion of hospitals, inpatient stays, and data elements varies in the different states. The documentation notes state-specific restrictions arising from confidentiality agreements related to hospitals, records, physicians, and discharges (for example, patients with HIV infections). The observational unit is the inpatient stay. Patient identifiers are not available. Diagnoses and procedures are coded with the ICD-9 Clinical Modification (ICD-9-CM). A maximum of 15 diagnosis codes and 15 procedure codes are allowed per inpatient stay. ICD-9-CM codes for external causes of injury and poisoning are stored separately. A maximum of four of those codes are allowed per inpatient stay. For this study, raw data for the calendar year 2006 were obtained from the AHRQ. These data were derived from 8,074,825 inpatients from 1,045 hospitals. The inpatients were admitted in 2006 or earlier and discharged in 2006.

Since 2002, German hospitals have been obligated to annually deliver a standard data set to the Institute for the Hospital Remuneration System (InEK). The data consist of information on all inpatients covered by the DRG system. Some of the aggregated data are published by the InEK for use in ecological studies. The data can further be used as a DRG statistic through teleprocessing at the Federal Statistical Office. In teleprocessing, a customer sends a script for a statistical software package to the Federal Statistical Office. The script is then executed by its staff and the resulting report is sent back to the customer. The observational unit in the DRG statistic is the inpatient stay. Data are available starting with the year 2005. No patient identifier is available. Diagnoses are coded with the ICD-10 German Modification (ICD-10-GM), and procedures with a national procedure classification. A maximum of 90 diagnosis codes and 101 procedure codes are allowed per inpatient stay. The inpatients included in this study were admitted in 2006 or earlier and discharged in 2006. The results are based either on the InEK data or on the DRG statistic for the calendar year 2006, both of which were published previously [14,15].

### Identification of the relevant ICD codes

A list of 502 ICD-10-GM codes indicating a possible ADE was previously developed [5]. The identified codes were classified into seven categories based on the validity of each one as an indicator of an ADE and its definition in the ICD-10:

● A.1: A drug-related causation was noted in the ICD-10, e.g., G44.4 “Drug-induced headache, not elsewhere classified”.

● A.2: A drug- or other substance-related causation was noted in the ICD-10, e.g., I42.7 “Cardiomyopathy due to drugs and other external agents”.

● B.1: The event was denoted as a drug poisoning, implying a non-physiological dosage, e.g., T36.0 “Poisoning: Penicillins”.

● B.2: The event was denoted as poisoning by or harmful use of a drug or other substance, e.g., T50.9 “Poisoning: Other and unspecified drugs, medicaments, and biological substances”.

● C: A drug-related causation was very likely, e.g., A04.7 “Enterocolitis due to Clostridium difficile”.

● D: A drug-related causation was likely, e.g., F52.2 “Failure of genital response”.

● E: A drug-related causation was possible, e.g., J81 “Pulmonary edema”.

Categories C, D, and E were distinguished through expert opinion [5]. The study only considered codes of categories A, B, and C, as they are the ones that very likely take into account the administration of a drug. For categories A.2 and B.2, however, other substances or measures may also have caused the event.

### Mapping process

The list of 502 ICD-10-GM codes indicating a possible ADE was mapped onto the ICD-10 NHS and the ICD-9-CM. Table 1 shows the result of the mapping process.

**Table 1:** Number of codes indicating an adverse drug event (ADE) in the ICD-10 NHS, ICD-10-GM, and ICD-9-CM

**Category**	**Definition**	**ICD-10 NHS (England)**	**ICD-10-GM 2006 (Germany)**	**ICD-9-CM 2006 (USA)**
A	Caused by a drug or other substance	122	179	275
*A.1*	*Caused by a drug*	*44*	*101*	*184*
*A.2*	*Caused by a drug or other substance*	*78*	*78*	*91*
B	Poisoning by or harmful use of a drug or other substance	189	148	232
*B.1*	*Poisoning by drug*	*133*	*133*	*195*
*B.2*	*Poisoning by or harmful use of a drug or other substance*	*56*	*15*	*37*
C	ADE very likely	27	30	18
A-C		338	357	525

For ICD-10 NHS mapping, a license was obtained from the WHO for the UK version of the ICD-10. Included with that license was a file from the NHS containing metadata of the ICD-10 NHS. After direct mapping from the ICD-10-GM to find the ADE codes in the ICD-10 NHS, the following aspects were determined: a) whether the resulting ICD-10 NHS codes truly indicated an ADE and b) whether the category of the resulting code was the same as the category from the starting code of the German ICD-10-GM. If necessary, the results were adapted accordingly. Furthermore, for those ADE codes of the German ICD-10-GM without a hit in the ICD-10 NHS, an appropriate mapping was determined manually, by browsing the list of codes in the ICD-10 NHS metadata. Open questions were resolved by a consensus reached by a group of two persons.

Documents for the ICD-9-CM were downloaded from the web page of the Centers for Disease Control and Prevention (cf. http://www.cdc.gov/nchs/icd/icd9cm.htm). Initially, automatic mapping was attempted using the different mapping tables: ICD-10-GM 2006 onto ICD-10 SGB-V 2.0 (an earlier German version of ICD-10) onto ICD-10 SGB-V 1.3 (an earlier German version of ICD-10) onto the German version of the ICD-9 WHO V6.0. The resulting ICD-9 WHO codes were then directly mapped onto the ICD-9-CM. Furthermore, the document of the ICD-9-CM, in rich text format (RTF), was manually checked for ADE codes using terms such as “drug”. The same aspects as described above for the mapping of ICD-10-GM onto ICD-10 NHS were determined and, as necessary, the results were adapted accordingly. Furthermore, for those ADE codes of the German ICD-10-GM without a hit in the ICD-9-CM, an appropriate mapping was determined, by browsing the RTF document. Open questions were resolved as described above.

### Data pre-processing

The data were cleaned to generate datasets comparable with the German DRG statistic, at least regarding basic plausibility checks. For the HES, episodes were aggregated to inpatient stays using the hospital provider spell number, sex, date of admission, method of admission, source of admission, and end of inpatient stay. Excluded were inpatients for whom data on age, sex, method of admission, destination after discharge, method of discharge, principal diagnosis, admission date, discharge date, or length of stay were missing or invalid. Also excluded were data from inpatients admitted before or discharged after the financial year, as well as duplicates. Thus, a total of 13,547,900 inpatient stays were included. For more than 7 million of them, a length of stay of null days was recorded. These inpatient stays were then excluded, based on the assumption that they were administratively recorded but not personally served by the hospitals. That kind of stay was not present in the datasets from Germany and the USA. Finally, 6,202,313 inpatient stays were considered (44.0% from the HES).

From the NIS inpatient stays were excluded in which data on sex, admission type, disposition of patient (discharge status), principal diagnosis, and length of stay were missing. Further excluded were data from inpatients admitted before 2006. The final number of inpatient stays considered was 7,125,028 (88.2% from the NIS).

Inpatients who underwent surgery were defined in the case of England by the value “one or more operative procedures carried out” as operation status code; in the case of Germany by at least one procedure code from the chapter "operation" of the national procedure classification; and in the case of the USA by at least one procedure code from those parts of the ICD-9-CM related to surgical interventions. All other cases were treated as not including an operation.

### Statistics

ADEs were identified using both the principal and the secondary diagnoses. If more than one ADE was identified for an individual inpatient stay, that stay was counted only once. The data were managed with MySQL 5.x (Oracle Corporation) and Microsoft Access 2007/2010. For analysis, Microsoft Excel 2007/2010 and SAS 9.2 (SAS Institute Inc.) were used. ADE rates were calculated by dividing the number of inpatient stays in which there was at least one ADE by the total number of inpatient stays. The rates are reported as percentages, with the 95% confidence interval (CI) in square brackets. Given the results available with the DRG statistic, the rates from England and the USA were adjusted for the distribution of the following data elements in the DRG statistic using a direct standardization: operation (yes/no), sex (male/female), emergency (yes/no), age at admission (≤ 53 years/>53 years). The mean age at admission for German inpatients was chosen as the threshold value for the age at admission. To correct for differences in coding completeness, the rates for each country were multiplied by the result of the “mean number of secondary diagnoses in Germany” divided by the “mean number of secondary diagnoses in this country”, yielding 1.71 for the HES, 1.00 for the DRG statistic, and 0.74 for the NIS.

To corrects for shifts of codes between subcategories occurring after the mapping, categories A.1 and A.2 were combined to form category A, and categories B.1 and B.2 to form category B. Inpatient stays were counted once within each remaining category (A, B, and C) for England and the USA. For Germany, inpatients stays were counted twice in categories A and B if an ADE occurred in each respective sub-category. Otherwise, they were counted once for the remaining categories (A, B, and C), as for England and the USA. Furthermore, the results for Germany were initially split into principal and secondary diagnoses. Among the patients in whom ADE was the principal diagnosis, 12% also had an ADE as the secondary diagnosis (unpublished results). To adjust for this overlap, the German prevalence rates for categories A, B, and C were multiplied by a factor of 0.88.

The odd ratios with their 95% CIs were calculated for operation status, sex, emergency status, age at admission, and length of stay (≤ 10 days/> 10 days). The five most frequent ADEs in each country are reported.

Due to the use of anonymized data in this study, the approval of an ethics committee was not necessary.

## Results

### Study populations

Table 2 shows the characteristics of the three study populations. Among the German population, there were fewer women (53.8%), fewer emergency cases (35.1%), and fewer operations (40.4%) than in the populations from England and the USA. German inpatients were older, with a mean age of 52.8 years (standard deviation 25.58 years, median 59 years), and were hospitalized longer, with a mean length of 7.5 days (standard deviation 8.79 days, median 5 days). There were noticeable differences between England and the USA regarding the frequencies of emergencies and operations. Thus, in the USA, inpatients were operated on more frequently (54.6% vs. 45.7%) and were less frequently admitted as emergency cases (45.7% vs. 54.2%).

**Table 2:** Characteristics of the study populations

	**England**	**Germany**	**USA**
Number of inpatients			
Total (%)	6,202,313 (100.0)	16,230,407 (100.0)	7,125,028 (100.0)
Male (%)	2,677,383 (43.2)	7,505,172 (46.2)	2,957,623 (41.5)
Emergency (%)	3,361,405 (54.2)	5,702,142 (35.1)	3,252,330 (45.7)
Operation (%)	2,836,784 (45.7)	6,563,549 (40.4)	3,890,723 (54.6)
Died (%)	208,596 (3.4)	388,682 (2.4)	143,279 (2.0)
Age in years			
Mean (std. dev.)	47.1 (28.27)	52.8 (25.58)	48.1 (27.90)
Median (Q1-Q3)	49 (25–72)	59 (35–73)	52 (27–72)
Length of stay in days			
Mean (std. dev.)	6.4 (12.34)	7.5 (8.79)	4.5 (6.08)
Median (Q1-Q3)	3 (1–6)	5 (X^†^-9)	3 (2–5)

^†^For reasons of confidentiality, this number was blinded by the German Federal Statistical Office.

### ADE prevalence

In the English HES, there were 142,845 inpatient stays related to ADEs (2.3% of 6,202,313 inpatient stays). For the German DRG statistic, there were 775,959 inpatient stays involving an ADE (4.8% of 16,230,407 inpatients) while according to the USA's NIS 520,555 inpatient stays involved an ADE (7.3% of 7,125,028 inpatient stays). The adjusted prevalence rate of ADEs was highest for the USA, with 5.64% [5.63–5.66%] followed by Germany with 4.78% [4.73–4.83%] (cf. Table 3). England had the lowest rate, with 3.22% [3.20–3.23%]. The rates in the three categories (A, B, and C) were comparable for Germany and the USA whereas in England the rate for ADEs in category A was significantly lower than that of either Germany or the USA: 0.99% [0.99–1.00%] vs. 3.14% [3.10–3.17%] and 4.43% [4.42–4.45%], respectively, and the rate for category B was twice as high, with 1.21% [1.20–1.22%]. The most frequent ADE in all countries was enterocolitis due to Clostridium difficile (category C), with 33,509 inpatients in England (23.5% of all inpatients with an ADE, prevalence rate 0.54%), 36,215 inpatients in Germany (4.7% of all inpatients with an ADE, prevalence rate 0.24%) [5], and 56,771 inpatients in the USA (10.9% of all inpatients with an ADE, prevalence rate 0.80%). The second most frequent ADE in Germany and the USA was secondary thrombocytopenia (category C). This was followed by drug poisoning (category B.1), both in England (rank 2–5) and in the USA (rank 3–5). The drugs specifically responsible were benzodiazepines, antidepressants, and nonsteroidal anti-inflammatory drugs. In Germany, drug-induced agranulocytosis and neutropenia (category A.1) and drug-induced aplastic anemia due to chemotherapy (category A.1) occupied positions 3 and 4 (cf. Table 4).

**Table 3:** Standardized prevalence rates of ADEs for England, Germany and the USA

		England^*^	**Germany**^**†**^	USA^*^
**Category**	**Definition**	**Rate in % [95% CI]**		
A	Caused by a drug or other substance	0.99 [0.99–1.00]	3.14 [3.10–3.17]	4.43 [4.42–4.45]
B	Poisoning by or harmful use of a drug or other substance	1.21 [1.20–1.22]	0.49 [0.48–0.51]	0.63 [0.63–0.64]
C	ADE very likely	1.07 [1.07–1.08]	1.14 [1.11–1.16]	0.86 [0.85–0.87]
A-C		3.22 [3.20–3.23]	4.78 [4.73–4.83]	5.64 [5.63–5.66]

^*^Inpatient stays were counted once within each category. The rates were adjusted for the distribution of the following data elements in the DRG statistic using a direct standardization: operation (yes/no), sex (male/female), emergency (yes/no), age at admission (≤ 53 years/> 53 years). The rates were further adjusted for the mean number of secondary diagnoses in Germany.

^†^Inpatients stays were counted twice in categories A and B if the ADE occurred in each of the sub-categories. Otherwise, they were counted once. To aggregate the initial results, which were differentiated according to principal and secondary diagnoses, the total number of inpatient stays for categories A, B, and C was multiplied by a factor of 0.88.

**Table 4:** The five most frequent ADE codes in England, Germany and USA, in descending order

**Code**	**Title**	**Category**	**Number of inpatients**
**Germany**^**†**^			
A04.7	Enterocolitis due to Clostridium difficile	C	36,215
D69.58	Other secondary thrombocytopenia, not classified as transfusion-refractory	C	32,600
D70.10	Drug-induced agranulocytosis and neutropenia: critical phase < 10 days	A.1	16,044
D61.10	Drug-induced aplastic anemia	A.1	14,002
D69.59	Secondary thrombocytopenia not otherwise specified	C	12,560
**England**			
A04.7	Enterocolitis due to Clostridium difficile	C	33,509
T39.1	Poisoning by 4-aminophenol derivatives	B.1	27,208
T43.2	Poisoning by other and unspecified antidepressants	B.1	8,231
T39.3	Poisoning by other nonsteroidal anti-inflammatory drug (NSAID)	B.1	8,051
T42.4	Poisoning by benzodiazepines	B.1	8,035
**USA**			
008.45	Intestinal infection due to Clostridium difficile	C	56,771
287.4	Secondary thrombocytopenia	C	22,700
969.4	Poisoning by benzodiazepine-based tranquilizers	B.1	12,683
969.0	Poisoning by antidepressants	B.1	7,332
965.4	Poisoning by aromatic analgesics, not elsewhere classified	B.1	5,119

^†^Translation from the German by the author.

### ADE associations

The odds ratios (ORs) for operation, sex, emergency, age at admission, and length of stay are presented in Table 5. In all countries, ADEs were associated with emergency cases (ORs between 1.391 [1.384–1.397] and 8.082 [8.065–8.099]) and less associated with surgical treatment (ORs between 0.367 [0.366–0.367] and 0.544 [0.543–0.545]) and length of stay ≤ 10 days (ORs between 0.340 [0.339–0.341] and 0.418 [0.416–0.420]). In England and Germany, ADEs were less associated with sex male (ORs = 0.810 [0.809–0.812] and 0.894 [0.890–0.898]) whereas in the USA the opposite was determined (OR = 1.225 [1.222–1.227]. In Germany and the USA, ADEs were less associated with age ≤ 53 years (ORs = 0.648 [0.645–0.651] and 0.842 [0.840–0.844]) whereas in England there was a slightly positive association (OR = 1.076 [1.073–1.078]).

**Table 5:** Odds ratios for ADEs in England, Germany, and the USA

**Variable**	**England**	**Germany**	**USA**
	**Odds ratio [95% CI]**		
Male	0.810 [0.809–0.812]	0.894 [0.890–0.898]	1.225 [1.222–1.227]
Emergency	8.082 [8.065–8.099]	1.391 [1.384–1.397]	2.336 [2.331–2.341]
Operation	0.367 [0.366–0.367]	0.591 [0.588–0.594]	0.544 [0.543–0.545]
Age ≤ 53 years	1.076 [1.073–1.078]	0.648 [0.645–0.651]	0.842 [0.840–0.844]
Length of stay ≤ 10 days	0.340 [0.339–0.341]	0.376 [0.374–0.378]	0.418 [0.416–0.420]

Confidence intervals are indicated in square brackets.

## Discussion

### Coding issues

To our knowledge, this is the first study presenting a transnational comparison of ADE rates based on routine data. ADE frequencies were comparable in Germany and the USA, with one out of 20 inpatients suffering an ADE at the time of admission or during the hospital stay. Patients with ADEs were older, were admitted as emergency cases, did not undergo any surgical procedure, and had a longer hospital stay. The majority of the ADEs could be identified by codes explicitly mentioning a drug. By contrast, the ADE prevalence rate in England was remarkably lower, with poisoning or harmful use coded more frequently than in Germany or the USA. More than 80% of the patients in England were admitted as emergencies. However, the five most frequent, very likely ADEs were nearly identical in England and the USA. Some of the differences between the three countries examined in this study might have been due to variations in documentation and coding. A report of the OECD highlighted those variations in comparing several of the characteristics of routine data for 19 countries. The mean number of secondary diagnoses for denominator cases of the AHRQ’s indicator “Foreign body left in during procedure” varied between 1.50 (Italy) and 6.72 (Belgium) [10]. The numbers for the USA and Germany were, respectively, 6.02 and 5.31 whereas in the UK the mean number of secondary diagnoses was 2.72. However, this does not explain the results of the current study, because the prevalence rates from England and the USA were adjusted for those differences. In the OECD’s report, six of the 17 countries for which information was available used a variant of the ICD-9 for coding; the remaining 11 used a variant of the ICD-10. Differences in the representation of specific diseases have been reported anecdotally between ICD-9 and ICD-10 but also between variants of ICD-10 [16]. Different code distributions for the five ADE categories of Germany’s ICD-10-GM and England’s ICD-10 and the USA's ICD-9-CM suggest classification differences as a possible reason for confounding, pointing out the value of explicitly addressing ADEs in the development of ICD-11.

### Comparison with the existing literature on ADE frequencies in routine data

Three previous studies used HES data to analyze the prevalence rate of ADEs [17-19]. Waller et al. reported a stable rate of inpatients with an admission due to an ADE of about 0.35% for the years 1996 to 2000 in England [18]. According to Patel et al., the prevalence rate for the years 1998 to 2005 was slightly higher, 0.50% [17]. Unlike the present work, neither study analyzed the association of ADEs with demographic or other characteristics. Waller et al. selected inpatient stays (“admissions”) as the observational unit, and Patel et al. episodes. The difference between the two studies might therefore have been due to the inclusion by Patel et al. of ADEs occurring during the hospital stay. In the third study, Wu et al. selected emergency admissions between 1999 and 2009, using all diagnoses in identifying ADEs and thus accepting ADEs occurring at the time of admission as well as later on [19]. Linking episodes to admissions, those authors found an overall prevalence rate for all years of 0.9% and a prevalence rate in the last year of their study (2009) of 1.1%. In contrast to this study, none of those three studies considered codes not explicitly mentioning drugs. Excluding category C from this study would have lowered the calculated rate by one third. The remaining unadjusted rate for England is within the published scope for HES data, thus underlining the appropriateness of the methodology applied herein. Unfortunately, comparable analyses for the USA, to the best of this author's knowledge, have not been published.

In a nationwide study in The Netherlands, a prevalence rate of 1.83% was reported for all acute non-planned inpatient stays in 2001 [20]. Corresponding with the results determined for Germany in the current study, patients suffering an ADE were older and more often female and had a longer mean length of stay (12.5 vs. 10 days). In an Australian study based on discharges from three hospitals over a period of three months in 2004 and applying the external causes codes of the ICD-10 Australian Modifications (ICD-10-AM), an ADR prevalence rate of 4.5% was reported [3]. This high prevalence rate likely reflects the comprehensive and well-established ADR reporting system of the three hospitals, as the authors concluded that “The ICD-10-AM coding surveillance is an effective and efficient method of improving ADR identification and reporting”. However, they noted that carrying out chart reviews of all marked cases was remarkably time-consuming.

### Differences in ADE frequencies between routine data and prospective monitoring studies

The frequency of ADEs and ADRs in hospitals as reported from prospective monitoring studies is higher than that determined by analyses of routine data. For example, in their meta-analysis of 39 studies Lazarou et al. estimated an ADR prevalence rate for the USA of 15.1% [21]. The studies analyzed by those authors reported a prevalence rate of serious ADEs of 1.0–16.8% (estimate 6.7%), thus demonstrating a relevant uncertainty regarding the “real” number. Indeed, one may ask whether ADEs are seriously under-reported in routine data. However, false positives have to be considered as well. Houghland et al. found that only 64.9% of ADEs indicated by an ICD-9-CM code could be confirmed through chart reviews [4]. Both under-reporting of ADEs because they have been incompletely documented and are therefore missing from routine data and over-reporting of ADEs because of false positives have to be taken into account. Alternatively, it may be the case that the two balance each other out, leading to plausible estimates. This latter notion is supported by evidence from other adverse events [22,23]. For example, a similar frequency of hospital-acquired pneumonia was determined by chart review and routine data analysis [22]. With chart review as the gold standard, routine data had a sensitivity of 43% and a positive predictive value of 64%, thereby equalizing an under- and an over-reporting. For pressure ulcer, a sensitivity of 47% was calculated in a comparison of routine data recorded in a quality management project with a cross-sectional validation survey [24]. The extrapolated period prevalence rate of 2.3% determined in the cross-sectional validation survey has to be compared with the rate of 1.4% of pressure ulcers detected through the analysis of routine data. In our study, the calculated rates of ADEs for England, Germany, and the USA were more similar than the ADE rates published from prospective monitoring studies.

### Study limitations

The routine data of England and the USA were adapted to the German data in order to reach a conformance level that excluded data quality issues, differences in demographics, and differences in service structure as possible confounders. In addition, NHS services uncommon in Germany and the USA were excluded. While the results are therefore to a large extent comparable, these adaptations may have introduced certain biases. Further research is necessary to weigh the pros and cons of the use of routine data in transnational health services research. On the one hand, it would be interesting to compare drug and medication safety during inpatient care, accepting that there are differences in the service structures of different countries. Thus, it may be that differences in ADE frequency highlight the influence of these different structures on ADE occurrence. On the other hand, drug and medication safety alone, independent of the service structure, may be of interest. In this case, a method would be needed to homogenize the data related to the service structure. Especially for England, the results presented here might not be representative of the HES database in full, given the exclusion of inpatient stays with a length of null days.

Differences in ADE frequencies arising from the different coding processes in the three countries were not considered in this study. For example, in Germany coding is often integrated into the clinical process whereas this might not be the case in England and USA. However, corrections for different levels of coding completeness may well have compensated for those factors.

The German data were available through teleprocessing only, which made the respective analysis much more difficult and resource-consuming than the direct use of raw data. Consequently, the German DRG statistic formed the basis of the transnational analysis. It was not feasible to consider questions arising from the HES and NIS analyses. Clearly, the mixture of classification revisions and national classification variants is problematic in the identification of ADEs using ICD codes. Thus, after mapping, there were shifts between categories (e.g., from A.1 to A.2) that occurred because of the slightly different denominations of a similar code. The results became increasingly incompatible at deeper category levels. For this reason, the ADE prevalence rates reported at the most detailed level were excluded.

The NIS uses a specific sample of hospitals in the USA [10], excluding, among others, the nationwide system of Veterans Hospitals. Furthermore, there are state-specific restrictions for the inclusion of inpatients or data sources. Nevertheless, the huge number of inpatients available in the NIS database should guarantee a reliable estimate of the ADE frequency in the USA.

In this study, there was an intentional underestimate of ADEs because of the focus on ICD codes with a close relationship to the administration of a drug, one either explicitly mentioned in the code or implicitly indicated by the disease. However, the list of codes applied here is the most complete one available [24]. In contrast to other publications, this list includes diseases that are pathognomonic for the administration of a drug, such that the ADE prevalence rate calculated from routine data provides a realistic picture of the true frequency of ADEs. ADEs of the type “need to add drug” and “untreated indication” were not included in the presented analysis. According to a recent study of emergency department patients, ADEs of those types account for 15% of all ADEs [25].

## Conclusions

Routine data are among the several readily available resources that can be used for health services research and to answer pharmaco-epidemiological questions. Pharmacovigilance based on spontaneous reporting by health professionals is extremely incomplete [26,27]. If used as trigger tool, routine data have the potential to achieve completeness. The prerequisite is coding in parallel with the inpatient stay. For the application in trigger tools routine data do not only include diagnoses but also lab values and other information [28]. However, from the point of view of transnational health services research, this is a goal for the remote future since it would first require an international standardization of terms beyond the diagnosis codes. In the meantime, it might be worthwhile to reach a worldwide consensus on a set of characteristics that should be provided in each publication on ADE frequencies, independent of the type of data.

## Competing interests

The author declares that he has no competing interests.

## Pre-publication history

The pre-publication history for this paper can be accessed here:

http://www.biomedcentral.com/1472-6963/14/125/prepub

## Data used in the manuscript

The Hospital Episodes Statistics (cf. http://www.hscic.gov.uk/hes) extract was purchased from the NHS Information Centre for Health and Social Care, for which a Data Re-Use Agreement (HES Extract Request NIC-40421-QRJ49) was signed by the author.

German analyses were based on data from the Research Data Center of both the national and the federal Offices of Statistics (cf. http://www.forschungsdatenzentrum.de/en/), the DRG statistic for the year 2006, and on the author's own calculations (contract ID ZD/44210010-10D055).

The Healthcare Cost and Utilization Project (HCUP) Nationwide Inpatient Sample (NIS) (cf. http://www.hcup-us.ahrq.gov/nisoverview.jsp) was purchased from the HCUP Central Distributor, under the auspices of the Agency for Healthcare Research and Quality (AHRQ). A Data Use Agreement was signed by the author.
